# Popularity and diversity: The negative relationship in baby names in the United Kingdom

**DOI:** 10.12688/f1000research.162476.1

**Published:** 2025-04-11

**Authors:** Yuji Ogihara

**Affiliations:** 1 Department of Psychology, College of Education, Psychology and Human Studies, Aoyama Gakuin University, Shibuya, Tokyo, 150-8366, Japan

**Keywords:** popularity, diversity, name, commonality, distribution

## Abstract

**Background:**

Previous research has shown that popular names have become less popular over time. Simultaneously, accumulated evidence has indicated that names have become more diverse. However, the association between these two phenomena was unclear. This association should be revealed for a better understanding of names and naming practices. Therefore, this study investigated the relationship between the popularity and diversity of names.

**Methods:**

I analyzed the data provided in a previous study in the U.K., which included complete records of all live births between 1996 and 2016 (N = 12,985,140).

**Results:**

I found that the correlations between diversity and popularity indicators were highly negative, showing that they are conceptually strongly related. This means that when diversity is high, popularity is low.

**Conclusions:**

Based on this study, we can predict one indicator from the other indicator. Because raw data on names are generally difficult to collect, this prediction is useful for understanding names and naming practices.

## Introduction

### Popularity of names has decreased

Previous research has indicated that popular names ^1^ have become less popular over time, suggesting that popularity of names has decreased (for a review, see Ogihara, 2025). For example, the rates of popular names decreased in the United States between 1880 and 2007 ( Twenge et al., 2010). In this study, the rates of the top 1, 10, 25, and 50 most popular names were calculated each year, and their historical changes were analyzed (also see Twenge et al., 2016). Furthermore, Bush et al. (2018) demonstrated that the rates of popular names (the top 1 and 10 most popular names) decreased in the United Kingdom (England and Wales) between 1996 and 2016 (also see Bush, 2020). Similar trends were reported in Germany ( Gerhards & Hackenbroch, 2000) and France ( Mignot, 2022).

Not only in the West (Europe and North America) but also in the East (Asia), this trend has been observed. For instance, Ogihara et al. (2015) showed that the rates of popular names (the top 1, 10, 20, and 50 most popular names) decreased in Japan between 2004 and 2013 (also see Ogihara, 2022). This shift has been consistently reported ( Ogihara, 2021; Ogihara & Ito, 2022; for a review, see Ogihara, 2025). In China ( Bao et al., 2021) and Indonesia ( Kuipers & Askuri, 2017), the popularity of names decreased as well.

### Diversity of names has increased

At the same time, emerging evidence has shown that the diversity of names has increased over time, showing that names have become more diverse. For example, Bush et al. (2018) indicated that the ratio of unique (distinctive) names (the relative value of name variety) increased in the U.K. between 1838 and 2016. Moreover, this trend was observed in the U.S. between 1880 and 2017 ( He, 2020; also see Mignot (2022) for a similar report in France).

Taken together, accumulated research has indicated that names have become less popular and more diverse. Bush et al. (2018) has shown that these two phenomena were simultaneously observed in the U.K. between 1996 and 2016, analyzing the same dataset.

### The relationship between popularity and diversity is unclear

However, the relationship between these two phenomena is unclear. Even though these two phenomena were reported within the same study ( Bush et al., 2018; Mignot, 2022), their relation was not directly investigated. Based on the meaning of the concepts (popularity and diversity), they are predicted to be negatively correlated. In other words, when the ratio of popular names is high, the diversity is expected to be relatively low. Similarly, when the ratio of popular names is low, the diversity is expected to be relatively high. Nevertheless, this prediction was not empirically tested. It is possible that even when the ratio of popular names is high, the diversity can also be high. For example, a population can be polarized, where some people give popular names, while others can give many varieties of unpopular names, leading to high popularity and diversity simultaneously.

This relationship between popularity and diversity should be uncovered. If the relationship is revealed, we can predict one indicator from the other. For instance, when the data and results for the top 10 most common names are available, we can infer its diversity from its popularity. In fact, this situation is frequently observed. Names are among the most private types of information. Thus, raw data on names is restricted from being openly shared, making it common for only the ranking of popular names (e.g., the top 10 most common names) to be disclosed (for a review, see Ogihara, 2025). Therefore, even when only one of the two indicators is available, we can estimate the distribution more precisely, which increases the understanding of the nature and phenomena of names and naming practices.

### The current study

Therefore, in this study, I examined the relationship between the popularity and diversity of names. Specifically, I analyzed the data in the U.K. presented in previous research ( Bush et al., 2018).

Based on the prior discussion above, it was predicted that there would be a negative correlation between the popularity and diversity indicators.

## Method

### Data

I analyzed the open data provided by Bush et al. (2018; Table S15 “Number of unique forenames, and forename diversity, in the Office for National Statistics dataset”). The data included variables on popular names and name diversity.

The original data is from the U.K. Office for National Statistics (2018), which included complete records of all live births in England and Wales for 21 years between 1996 and 2016. A total of 12,985,140 names were recorded, with an average of 618,340 names per year. It should be noted that names with a count of 2 or 1 were redacted to protect the confidentiality of individuals ( Office for National Statistics, 2018).

### Indicator

**Popularity.** As a popularity indicator, I used the variables “% of birth records registered with the most popular name” and “% of birth records accounted for by the top 10 names” ( Bush et al., 2018, Table S15). These indicators have been used in many prior studies (e.g., Bush, 2020; Kuipers & Askuri, 2017; Mignot, 2022; Ogihara et al., 2015; Ogihara, 2022; Twenge et al., 2010, 2016).

**Diversity.** As a diversity indicator, I used the variable “Forename diversity (i.e., ratio of the no. of unique forenames to the total no. of birth records per year)” ( Bush et al., 2018, Table S15). Thus, this indicator represents the relative value of name variety. For example, when this value is 0.01, it means that there are 10 name types among 1,000 people. When this value is high, diversity is also high (there are more name types, meaning that the group is more diverse). This indicator has been used in previous research (e.g., He, 2020).

## Results

Simple Pearson’s correlation coefficients among the year, popularity indicators, and diversity indicator are summarized in Table 1.

**Table 1.** Simple Pearson’s correlation coefficients.

	Year	Popularity (top 1)	Popularity (top 10)	Diversity
Year	-	−.955	−.973	.979
Popularity (top 1)	−.955	-	.969	−.960
Popularity (top 10)	−.973	.969	-	−.994
Diversity	.979	−.960	−.994	-

### Relationship within popularity indicators

The ratio of the most popular name and the ratio of the top 10 most popular names were strongly correlated, *r* = .969. This result means that these two indicators consistently measure the same concept, increasing the validity of these two indicators as name popularity indices.

### Relationship between popularity and diversity

**Figure 1.**
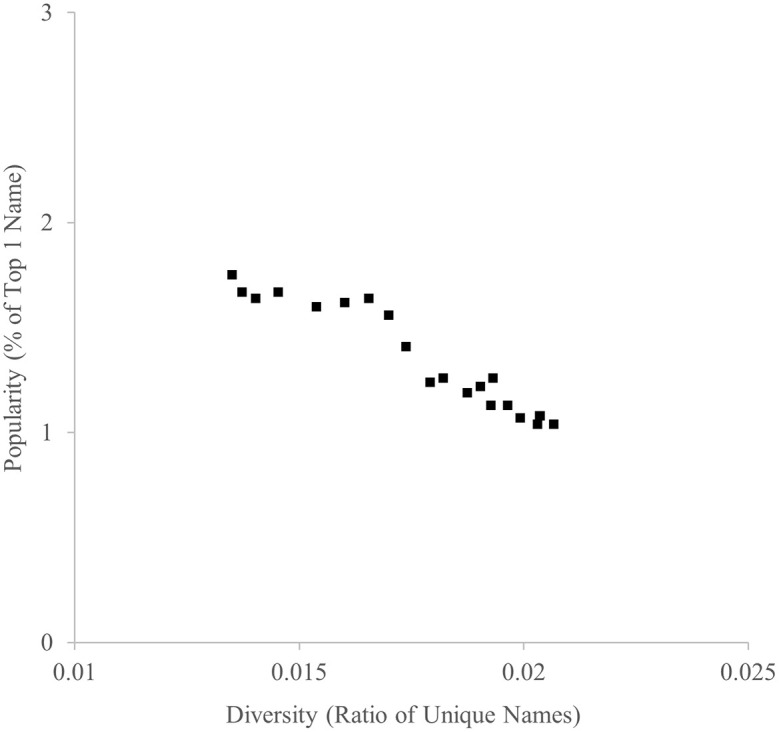
Diversity (ratio of unique names) and popularity (% of top 1 name) indicators of baby names in the U.K., 1996-2016.

The ratio of unique names and the ratio of the most popular name between 1996 and 2016 are indicated in Figure 1. As predicted, they were highly negatively correlated, *r* = −.960.

**Figure 2.**
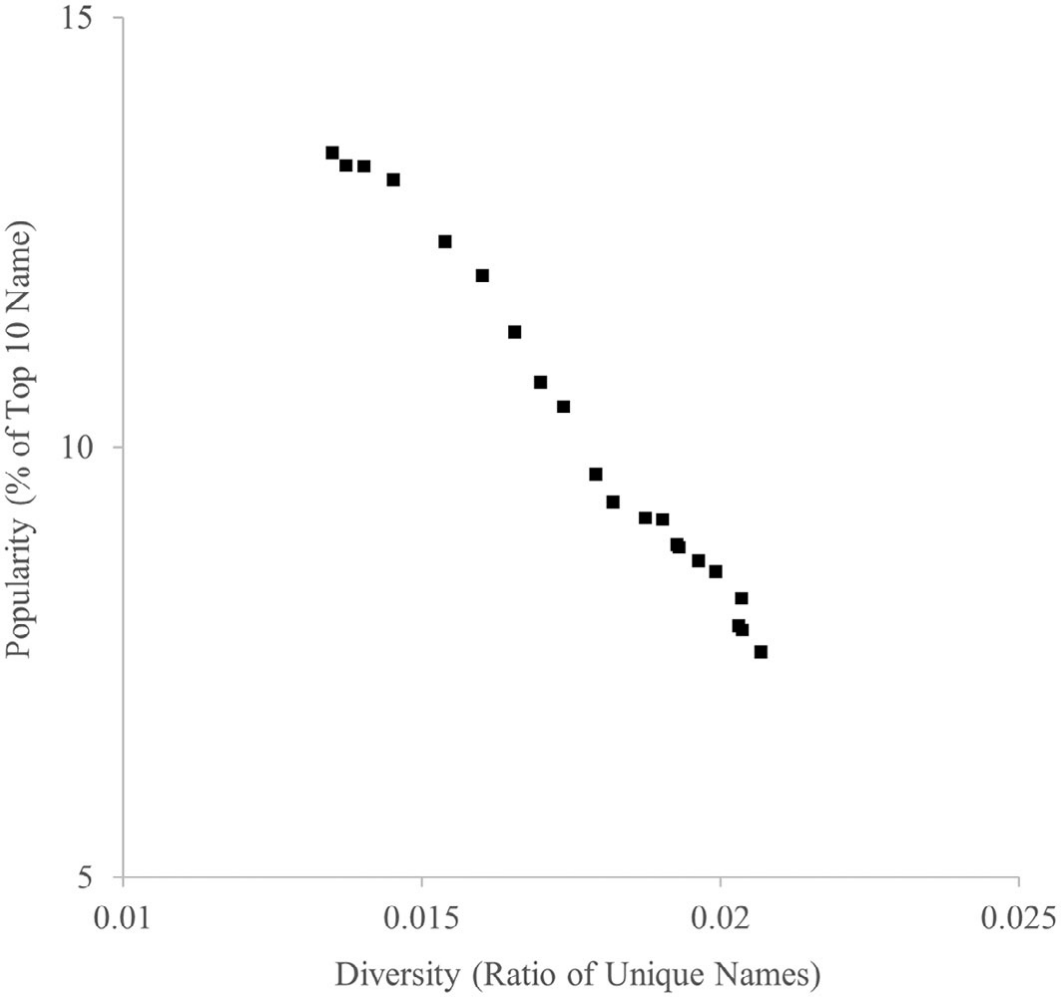
Diversity (ratio of unique names) and popularity (% of top 10 names) indicators of baby names in the U.K., 1996-2016.

Similarly, the ratio of unique names and the ratio of the top 10 most popular names are indicated in Figure 2. They were also highly negatively correlated, *r* = −.994.

## Discussion

Previous research has shown that names have become less popular over time (for a review, see Ogihara, 2025). At the same time, accumulated evidence has indicated that names have become more diverse over time. However, the association between these two phenomena was unclear. This association should be revealed for a better understanding of names and naming practices. Therefore, this study investigated the relationship between the popularity and diversity of names.

I analyzed the name data provided by the previous study in the U.K. ( Bush et al., 2018). I found that the correlations between the diversity and popularity indicators were highly negative, showing that they are conceptually strongly related. Specifically, in years when the ratio of unique names was high, the ratios of popular names were low. This means that when diversity is high, popularity is low. This association was very strong in the current dataset.

Based on this study, we can predict one indicator from the other indicator. We can infer diversity from popularity or popularity from diversity. Because raw data on names are generally difficult to collect, this prediction is useful for understanding names and naming practices.

### Limitation and future direction

This study examined the relationship between diversity and popularity of names in the U.K. (England and Wales), which showed highly negative associations between them. Nevertheless, it is unclear whether this relationship is observed in other nations. Names are cultural products and are affected by many factors (e.g., Morling, 2016; Morling & Lamoreaux, 2008). Thus, it is necessary to investigate this relationship in other nations.

## Author contributions

The author confirms being the sole contributor of this work and approved it for publication.

## Ethics and consent

Ethical approval and consent were not required.

## Data Availability

I analyzed the open data provided by Bush et al. (2018; Table S15 “Number of unique forenames, and forename diversity, in the Office for National Statistics dataset”).

## Footnotes

^1^

In this article, I use the term “names” to refer to “first names” (given names, personal names, forenames), not “last names” (family names, surnames).
