# Supplementary material for: Popularity and diversity: The negative relationship in baby names in the United Kingdom
Source: F1000Res. 2025 Dec 24;14:424. Originally published 2025 Apr 11. [Version 3] doi: 10.12688/f1000research.162476.3 (PMC12822686; doi:10.12688/f1000research.162476.3)
Supplement: Peer review history

# Reviewer report: Reviewer response for version 3

**DOI:** 10.5256/f1000research.194130.r444491

**Published:** 2026-01-07

**Authors:** Han-Wu-Shuang Bao

**Affiliations:** 1 School of Psychology and Cognitive Science, East China Normal University, Shanghai, Shanghai, China

I would like to thank the author for addressing my concern by reporting partial correlations in the new section, "Controlling for a possible confounding factor (year)". Surprisingly, however, none of the results reported in this article include statistical significance information, such as *p* values. While the simple correlations are close to 1 and are undoubtedly significant, the significance of the partial correlations (given the sample size of 21 years) is unclear. My calculations based on *r* and *n* suggest that *r* = −.887 and *r* = .577 are significant at *p* < .05, but *r* = −.419 has a *p* value of .06. I would therefore recommend that the author reports the exact *p* values in order to reach a more scientifically sound conclusion.

Is the work clearly and accurately presented and does it cite the current literature?

Yes

If applicable, is the statistical analysis and its interpretation appropriate?

No

Are all the source data underlying the results available to ensure full reproducibility?

Yes

Is the study design appropriate and is the work technically sound?

No

Are the conclusions drawn adequately supported by the results?

Partly

Are sufficient details of methods and analysis provided to allow replication by others?

Yes

Reviewer Expertise:

cultural change, names, naming practices, name uniqueness

I confirm that I have read this submission and believe that I have an appropriate level of expertise to confirm that it is of an acceptable scientific standard, however I have significant reservations, as outlined above.

# Reviewer report: Reviewer response for version 3

**DOI:** 10.5256/f1000research.194130.r444492

**Published:** 2026-01-02

**Authors:** Basil Osayin Daudu

**Affiliations:** 1 Kogi State University, Anyigba, Kogi, Nigeria

I have no further comments to make.

Is the work clearly and accurately presented and does it cite the current literature?

Yes

If applicable, is the statistical analysis and its interpretation appropriate?

Yes

Are all the source data underlying the results available to ensure full reproducibility?

Yes

Is the study design appropriate and is the work technically sound?

Partly

Are the conclusions drawn adequately supported by the results?

Yes

Are sufficient details of methods and analysis provided to allow replication by others?

Partly

Reviewer Expertise:

Philosophy, AI, African Studies, Health

I confirm that I have read this submission and believe that I have an appropriate level of expertise to confirm that it is of an acceptable scientific standard.

# Reviewer report: Reviewer response for version 2

**DOI:** 10.5256/f1000research.184627.r438916

**Published:** 2025-12-31

**Authors:** Süleyman Kasap

**Affiliations:** 1 Van Yüzüncü Yıl University, Bardakçı, Turkey

The manuscript investigates the correlation between two well-established demographic trends: the decreasing popularity of common names and the increasing diversity of names. Using a large, pre-existing dataset from the U.K., the author reports near-perfect negative correlations between measures of name popularity (top 1 and top 10 name shares) and a measure of name diversity (ratio of unique names). The central claim is that these indicators are so strongly related that one can be predicted from the other, offering a practical tool for researchers with limited data access.

 While the topic is relevant to onomastics and cultural sociology, the manuscript in its current form makes a negligible contribution to the literature. The core finding—that measures of concentration and diversity in a finite distribution are inversely related—is a statistical tautology, not a novel empirical discovery. The study lacks methodological rigor, a clear theoretical framework, and a meaningful interpretation of results. The manuscript reads more as a replication or secondary data analysis note than as a standalone research article.

 1. **Lack of Novelty and Conceptual Contribution:**

 The introduction establishes that prior research has documented *both* decreasing popularity and increasing diversity over time. The stated research gap is that the "relationship between these two phenomena is unclear." However, this relationship is logically and mathematically inherent. If the share of births accounted for by the top names decreases, the remaining share must be distributed among a larger set of less common names, inherently increasing diversity metrics (like the unique name ratio used here). The analysis merely quantifies this inherent relationship within one dataset. The manuscript fails to articulate what new sociological, psychological, or cultural insight is gained from this quantification.

 2. **Methodological and Statistical Concerns:** The chosen indicators are not independent. The "ratio of unique names" and the "percentage accounted for by the top N names" are two sides of the same coin when analyzing a single, bounded distribution (all births in a year). A high concentration in the top names *necessarily* limits the potential for a high unique name ratio, and vice-versa. The near-perfect correlations (r = -0.96, -0.99) are therefore expected and do not reveal a "conceptual" relationship but a mathematical one.  

 Lack of Statistical Control: The entire analysis is based on bivariate correlations across 21 time points. The strong negative correlations are almost certainly inflated by a strong shared time trend (both measures show clear linear trends over time, as evidenced by the high negative correlations with "Year" in Table 1). A partial correlation controlling for year, or a time-series analysis that accounts for autocorrelation, is essential to determine if a relationship exists beyond the simultaneous secular trends. The current analysis risks being spurious.

 Insufficient Detail: The manuscript lacks a clear operational definition of the key variables in the results section. The exact calculation of the "ratio of unique names" is critical. Is it (number of distinct names) / (total births)? If so, this is extremely sensitive to the recording of very rare names and the data redaction policy (names with count ≤2 removed), which is a significant limitation not adequately discussed.

 Weak Theoretical Framework and Interpretation: The introduction reviews literature on trends but does not build a theoretical argument for why testing this correlation is meaningful. The discussion does not interpret the results beyond restating them. The proposed "utility"—predicting one indicator from another—is overstated. Given the mathematical linkage, if a researcher has the share of the top 10 names, they can already make a strong bounded estimate of diversity without needing this specific correlation equation from U.K. data. The claim that this helps when "raw data...is restricted" is weak, as the prediction requires precisely the kind of summarized data (top N shares) that is already publicly available.

Is the work clearly and accurately presented and does it cite the current literature?

No

If applicable, is the statistical analysis and its interpretation appropriate?

No

Are all the source data underlying the results available to ensure full reproducibility?

Partly

Is the study design appropriate and is the work technically sound?

No

Are the conclusions drawn adequately supported by the results?

Partly

Are sufficient details of methods and analysis provided to allow replication by others?

No

Reviewer Expertise:

Linguistics , onomastics

I confirm that I have read this submission and believe that I have an appropriate level of expertise to state that I do not consider it to be of an acceptable scientific standard, for reasons outlined above.

## References

[1] : Impact of bilingualism and the difficulties of having minority-specific names in another dominant society: Turkish context for minority Kurdish society. *Onoma* .2021;56: 10.34158/ONOMA.56/2021/9 167-186 10.34158/ONOMA.56/2021/9

# Reviewer report: Reviewer response for version 2

**DOI:** 10.5256/f1000research.184627.r431893

**Published:** 2025-11-26

**Authors:** Basil Osayin Daudu

**Affiliations:** 1 Kogi State University, Anyigba, Kogi, Nigeria

The paper is an interesting one. The author uses infographics (figures, tables, and illustrations) of some existing literature to argue for the correlations between the popularity and diversity of names in the UK, as one indicator predicts the other and vice versa.

 However, some areas of concern need clarification:

 1. Starting with the abstract, the problem, the objective results, and conclusions of the study are clearly stated, but the methodology lacks detailed information on the analysis of the available material used.

 2. The uncertainty of the popularity and diversity of baby names, which the author also acknowledged. Adding my voice, “name” and “naming practice” may be interrelated, but they are distinct concepts of investigation. This differs from one culture to another. Taking cognizance of the study’s case study “United Kingdom” (England and Wales) as cited in the sixth line of the first paragraph of the introduction, Scotland and Northern Ireland are also considered as parts of the United Kingdom. So, these countries may have some shared similarities in name and naming practices, but they are not basically one and the same due to certain factors. This clearly shows how broad the scope of the study is, which largely contributes to the many uncertainties and predictions (not certainty) in the study. Therefore, narrowing down the scope to one of the countries in the United Kingdom would have produced better and more convincing results.

 3. The literature is very rich and up-to-date. My concern here is that most of the literature is largely by Ogihara and Bush et al. If we were to go by 4% single citation authorship, this paper would have been outrightly rejected. The author, too, perceives this lapse and therefore acknowledges the lack of sufficient material for the study.

 4. A more disturbing aspect of the study is the author’s adoption of Bush et al.’s study of records of all live births between 1996 and 2016. Another study by Bush et al., between 1838 and 2016. Now, if accepted the way it is, the problem of the study will be that of defining the scope. And to address this issue, the paper should preferably be titled “A Review… (Or similar title)” to fully communicate the author’s claims and evidence.

**Decision:**

 I really commend the author for his use of statistical techniques and infographics in his work. The work is good in the sense that it is a representation of existing literature in which the author lends his voice to the discourse. But reframing the title to correctly capture the content of the work would resolve a whole lot of uncertainties. In this regard, the author is in a better position to do it.

Is the work clearly and accurately presented and does it cite the current literature?

Yes

If applicable, is the statistical analysis and its interpretation appropriate?

Yes

Are all the source data underlying the results available to ensure full reproducibility?

Yes

Is the study design appropriate and is the work technically sound?

Partly

Are the conclusions drawn adequately supported by the results?

Yes

Are sufficient details of methods and analysis provided to allow replication by others?

Partly

Reviewer Expertise:

AI, African Studies, Health

I confirm that I have read this submission and believe that I have an appropriate level of expertise to confirm that it is of an acceptable scientific standard, however I have significant reservations, as outlined above.

# Reviewer report: Reviewer response for version 2

**DOI:** 10.5256/f1000research.184627.r398444

**Published:** 2025-09-02

**Authors:** Stephen J. Bush

**Affiliations:** 1 Xi'an Jiaotong University, Xi'an, Shaanxi, China

While I remain broadly in favour of this work and its utility in facilitating further research, as far as I can tell, the revised version of this article differs only minimally from the original. Specifically, the inclusion of two clarifying footnotes and a ‘limitations’ statement regarding the original data not distinguishing between boys’ and girls’ names.

 While I appreciate these additions, many of the points raised in the original reviews with regard to alternative or supporting analyses have not been addressed in the text – although could conceivably have strengthened the work further.

Is the work clearly and accurately presented and does it cite the current literature?

Yes

If applicable, is the statistical analysis and its interpretation appropriate?

Yes

Are all the source data underlying the results available to ensure full reproducibility?

Yes

Is the study design appropriate and is the work technically sound?

Partly

Are the conclusions drawn adequately supported by the results?

Partly

Are sufficient details of methods and analysis provided to allow replication by others?

Yes

Reviewer Expertise:

UK naming trends and practices

I confirm that I have read this submission and believe that I have an appropriate level of expertise to confirm that it is of an acceptable scientific standard, however I have significant reservations, as outlined above.

# Reviewer report: Reviewer response for version 2

**DOI:** 10.5256/f1000research.184627.r398443

**Published:** 2025-08-22

**Authors:** Han-Wu-Shuang Bao

**Affiliations:** 1 School of Psychology and Cognitive Science, East China Normal University, Shanghai, Shanghai, China

Thanks for the author's responses to my comments. I feel satisfied with his thoughts on the 2nd and 3rd points, but I still suggest the author reconsidering the 1st point with some further analyses, perhaps presented in footnotes. I agree that it was not necessary to determine which variable precedes the other, but even if the conclusion is purely correlational, it might be spurious due to a thrid confounder. A simple correlation between A and B might be high and significant merely due to a third variable C if both A and B are correlated with C. In this situation, it would not be appropriate to conclude that A is associated with B, because their association (correlation or "relationship") might disappear when C is considered. This is why social sciences usually consider partial correlations and multiple regression analyses to partial out confounders, and why time series analyses usually account for a common time trend or each variable's autocorrelation. Here, in this article, some conclusions about the "relationship between popularity and diversity" (e.g., "they are conceptually strongly related") require such a more rigorous test. Otherwise, if one found a strongly positive correlation between life expectancy and name diversity over time (which is highly possible if they change linearly), it might also be concluded that "they are conceptually strongly related" and even "to predict one indicator from the other indicator", which are not proper because time is the confounder underlying the two variables. Therefore, I still recommend that the author consider at least one more rigorous test beyond a simple correlation to empirically address this point. A practical one is to simply control for year and report their partial correlation.

Is the work clearly and accurately presented and does it cite the current literature?

Yes

If applicable, is the statistical analysis and its interpretation appropriate?

No

Are all the source data underlying the results available to ensure full reproducibility?

Yes

Is the study design appropriate and is the work technically sound?

No

Are the conclusions drawn adequately supported by the results?

Partly

Are sufficient details of methods and analysis provided to allow replication by others?

Yes

Reviewer Expertise:

cultural change, names, naming practices, name uniqueness

I confirm that I have read this submission and believe that I have an appropriate level of expertise to confirm that it is of an acceptable scientific standard, however I have significant reservations, as outlined above.

# Reviewer report: Reviewer response for version 1

**DOI:** 10.5256/f1000research.178684.r378193

**Published:** 2025-05-29

**Authors:** Stephen J. Bush

**Affiliations:** 1 Xi'an Jiaotong University, Xi'an, Shaanxi, China

This is a short report which reanalyses previously published UK name data to demonstrate an inverse relationship between name popularity and name diversity, such that one variable could be used predict the other. This could provide a useful platform for future name research as many name datasets are restricted to either the top X names per year or otherwise redact rare names (about which, more below); that is, it is only practically possible to derive the first variable from them (‘popularity’). In this respect, these results may facilitate the wider interpretation of smaller, and potentially overlooked, name datasets (e.g. ‘top 10’ lists).

 Nevertheless, the result remains a single, simple, correlation analysis. Beyond the suggestions made by the first reviewer (with which I concur), the conclusion may be strengthened by some corroboratory analyses, for instance using an independent dataset sampling the same population, and/or using name datasets that do not redact rarer names (which would otherwise affect the calculations).

 It is important to note that the raw data used for the analysis (Table S15 of Bush et al. 2018) ultimately derives from birth registration counts compiled by the UK Office for National Statistics (ONS); specifically, https://www.ons.gov.uk/file?uri=/peoplepopulationandcommunity/birthsdeathsandmarriages/livebirths/datasets/babynamesenglandandwalesbabynamesstatisticsboys/2016/adhocallbabynames1996to2016.xls, and strictly speaking covering only England and Wales rather than the entire UK. As with many name datasets, the long tail of the name distribution is redacted: in this case, names registered to < 3 people per year are excluded. As such, for any given year, the “diversity indicator” (the ratio of the no. of unique forenames to the total number of birth records per year) will be under-estimated. In addition, because the total no. of birth records will be lower than the total number of births, the “popularity indicator” (% of birth records registered with a top 1/top 10 name) will be over-estimated.

 For instance, we can see from Table S15 that in 2016, the total number of birth records was 639,126 (this number being used to calculate both the popularity and diversity indicators). However, the total number of births in 2016 was 696,271, an approx. 9% difference (this value is also made available by the ONS but in a separate file; see the link to ‘download figure 1 data’ in https://www.ons.gov.uk/peoplepopulationandcommunity/birthsdeathsandmarriages/livebirths/bulletins/birthsummarytablesenglandandwales/2023#live-births).

 It would bolster the overall conclusion – that popularity and diversity are negatively correlated – if it could be demonstrated that refinements to these values make negligible difference. A simple way of doing so would be to calculate them using birth registration datasets that do not redact rare names in the first place. Examples present themselves in the form of data from the Government of Alberta, Canada, specifically for the years 1980-2020 (see https://open.alberta.ca/opendata/frequency-and-ranking-of-baby-names-by-year-and-gender; specific file: https://open.alberta.ca/dataset/11245675-b047-49fc-8bd1-cc2ce8314a6d/resource/e8aac308-c754-484c-b446-0c57ed0e8d37/download/baby-names-frequency_1980_2020.xlsx) and, to draw the focus back to the UK, data from the National Records of Scotland, covering the years 1974-2023 ( https://www.nrscotland.gov.uk/media/mbmjzs2q/full-list-1974-2023.csv).

 In addition, two points foundational to the overall argument – that “names have become less popular over time” and that “raw data on names is restricted from being openly shared, making it common for only the ranking of popular names” are supported by reference to the author’s not-yet-published review (Ogihara 2025 ‘Uncommon names are increasing globally’). To provide further context, it would help if a citation was provided for at least a pre-print of this manuscript. I am particularly drawn to the point that names have become less popular over time as, in the UK, this most strongly manifests as the 20 ^th^ century progresses, i.e. during the time period covered by the ONS data. It would therefore be useful to know to what extent the relationship of popularity and diversity holds over a longer time period, and in that respect whether the conclusion can be generalised. That in mind, Bush et al. 2018 contains two different datasets sampling birth records from the English and Welsh population, of which the present work uses only one of them (from Table S15; the ONS dataset, which is much greater in depth but narrower in temporal scope). The second dataset – which has the same popularity and diversity indicators – is available in Table S6, and may be viewed as a representative population sample from 1838-2014, albeit of varying coverage depths per year. Nevertheless, a cursory examination of this data suggests that, as with the ONS data, there is a strongly negative correlation of popularity and diversity for the years 1996-2010 (this time period chosen because within it there are > 10,000 birth records per year). If we accept this as independent support of the conclusion drawn using the ONS data (and thereby the utility of this dataset), then it may be worth looking at how popularity and diversity correlate throughout history, too. This is of particular relevance as the conclusion drawn in the abstract (“we can predict one indicator from the other indicator”) implies a general relationship between the concepts that may not be universally true and that instead they may only “consistently measure the same concept” for a limited period of time.

 I have expanded the Table S6 dataset substantially since the original publication; see Bush 2024 ( https://doi.org/10.5195/names.2024.2543) and its raw data, specifically https://github.com/sjbush/uk_bmd/blob/main/dataset_B/summary_of_records_per_year.txt, which is essentially equivalent to Table S15 and contains the columns “most popular forename (% of records)” and “forename diversity (i.e., ratio of the no. of different forenames to the total no. of records per year)”. It should be clear from this data that before (approx.) the second half of the 20 ^th^ century, and compared to contemporary records, forename diversity was not only persistently lower but that popular names were given to a higher proportion of people and remained popular for longer, e.g. John and Mary were consistently the most popular male and female names, respectively, for a century (see https://demos.flourish.studio/namehistory/?names=John,Mary, which visualises data from Bush et al. 2018). So, although the present work (using late-20th century data) finds that one indicator predicts the other, it also appears the case that (earlier in the 20th century) popularity could vary without discernible change to diversity. Consequently, the two variables do not necessarily change simultaneously. This may be the subject of a more in-depth analysis but could be usefully commented upon here.

 Finally, could some comment also be made on the gender of names, and the effect this may have on the conclusions? In contemporary UK name data, there is typically a larger pool of female than male names (discussed in Bush 2020, https://doi.org/10.1080/00277738.2020.1775471): that is, one would expect female names to have a higher “diversity indicator” and lower “popularity indicator” than male names. Accordingly, a direct correlation of popularity with diversity may not quite be comparing like with like: diversity being affected more strongly by female names, but popularity by male (such that in Table S15, the top 1 names, across the whole population, are all male).

Is the work clearly and accurately presented and does it cite the current literature?

Yes

If applicable, is the statistical analysis and its interpretation appropriate?

Yes

Are all the source data underlying the results available to ensure full reproducibility?

Yes

Is the study design appropriate and is the work technically sound?

Partly

Are the conclusions drawn adequately supported by the results?

Partly

Are sufficient details of methods and analysis provided to allow replication by others?

Yes

Reviewer Expertise:

UK naming trends and practices

I confirm that I have read this submission and believe that I have an appropriate level of expertise to confirm that it is of an acceptable scientific standard, however I have significant reservations, as outlined above.

# Response

**Published:** 2025-07-06

**Authors:** Yuji Ogihara

**Affiliations:** Psychology, Aoyama Gakuin Daigaku, Shibuya, Tokyo, Japan

July 6th, 2025

 Dear Dr. Stephen J. Bush,

 Thank you very much for reviewing my manuscript and providing valuable comments.

 I have modified the manuscript extensively according to the reviewers’ comments.

 I offer my responses to each comment below.

 I have copied and pasted all of your comments without making changes.

***This is a short report which reanalyses previously published UK name data to demonstrate an inverse relationship between name popularity and name diversity, such that one variable could be used predict the other. This could provide a useful platform for future name research as many name datasets are restricted to either the top X names per year or otherwise redact rare names (about which, more below); that is, it is only practically possible to derive the first variable from them (‘popularity’). In this respect, these results may facilitate the wider interpretation of smaller, and potentially overlooked, name datasets (e.g. ‘top 10’ lists).***

 Thank you for your summary of this article and your words of praise.

***Nevertheless, the result remains a single, simple, correlation analysis. Beyond the suggestions made by the first reviewer (with which I concur), the conclusion may be strengthened by some corroboratory analyses, for instance using an independent dataset sampling the same population, and/or using name datasets that do not redact rarer names (which would otherwise affect the calculations).***

 I appreciate your constructive comment. As I replied to the first reviewer, I basically agree with your opinion. Yet, at least in this article, I would like to focus on the dataset used in a previous study (details are explained below).

***It is important to note that the raw data used for the analysis (Table S15 of Bush et al. 2018) ultimately derives from birth registration counts compiled by the UK Office for National Statistics (ONS); specifically, https://www.ons.gov.uk/file?uri=/peoplepopulationandcommunity/birthsdeathsandmarriages/livebirths/datasets/babynamesenglandandwalesbabynamesstatisticsboys/2016/adhocallbabynames1996to2016.xls, and strictly speaking covering only England and Wales rather than the entire UK. As with many name datasets, the long tail of the name distribution is redacted: in this case, names registered to < 3 people per year are excluded. As such, for any given year, the “diversity indicator” (the ratio of the no. of unique forenames to the total number of birth records per year) will be under-estimated. In addition, because the total no. of birth records will be lower than the total number of births, the “popularity indicator” (% of birth records registered with a top 1/top 10 name) will be over-estimated.***

***For instance, we can see from Table S15 that in 2016, the total number of birth records was 639,126 (this number being used to calculate both the popularity and diversity indicators). However, the total number of births in 2016 was 696,271, an approx. 9% difference (this value is also made available by the ONS but in a separate file; see the link to ‘download figure 1 data’ in https://www.ons.gov.uk/peoplepopulationandcommunity/birthsdeathsandmarriages/livebirths/bulletins/birthsummarytablesenglandandwales/2023#live-births).***

 Thank you for your valuable note. I agree with you that this point is important.

 The previous version of the article already stated that the data covered only England and Wales rather than the entire UK and that the long tail of the name distribution was redacted, as follows.

 “The original data is from the U.K. Office for National Statistics (2018), which included complete records of all live births in England and Wales for 21 years between 1996 and 2016. A total of 12,985,140 names were recorded, with an average of 618,340 names per year. It should be noted that names with a count of 2 or 1 were redacted to protect the confidentiality of individuals (Office for National Statistics, 2018).” (Data section)

 Thus, I have added the possible differences of the two indicators in the revised article, as follows.

 “Due to the redaction of names with a count of 2 or 1, the total number of birth records (denominator) is lower than the total number of births. In contrast, the top 1 and 10 most popular names (numerator) are not influenced by the redaction. As a result, this popularity indicator is higher than the actual popularity.” (Footnote 2)

 “Due to the redaction of names with a count of 2 or 1, the total number of birth records (denominator) is lower than the total number of births. In contrast, the number of unique forenames (numerator) is also lower than the total number of unique forenames. Thus, this diversity indicator is different from the actual diversity, but its direction (lower or higher) is difficult to predict.” (Footnote 3)

***It would bolster the overall conclusion – that popularity and diversity are negatively correlated – if it could be demonstrated that refinements to these values make negligible difference. A simple way of doing so would be to calculate them using birth registration datasets that do not redact rare names in the first place. Examples present themselves in the form of data from the Government of Alberta, Canada, specifically for the years 1980-2020 (see https://open.alberta.ca/opendata/frequency-and-ranking-of-baby-names-by-year-and-gender; specific file: https://open.alberta.ca/dataset/11245675-b047-49fc-8bd1-cc2ce8314a6d/resource/e8aac308-c754-484c-b446-0c57ed0e8d37/download/baby-names-frequency_1980_2020.xlsx) and, to draw the focus back to the UK, data from the National Records of Scotland, covering the years 1974-2023 (https://www.nrscotland.gov.uk/media/mbmjzs2q/full-list-1974-2023.csv).***

 I appreciate your valuable comment. Datasets that do not redact rare names are especially precious because such names are frequently excluded.

 Yet, this article focuses on names from England and Wales. Thus, I would like to examine these datasets in future research.

***In addition, two points foundational to the overall argument – that “names have become less popular over time” and that “raw data on names is restricted from being openly shared, making it common for only the ranking of popular names” are supported by reference to the author’s not-yet-published review (Ogihara 2025 ‘Uncommon names are increasing globally’). To provide further context, it would help if a citation was provided for at least a pre-print of this manuscript. I am particularly drawn to the point that names have become less popular over time as, in the UK, this most strongly manifests as the 20th century progresses, i.e. during the time period covered by the ONS data. It would therefore be useful to know to what extent the relationship of popularity and diversity holds over a longer time period, and in that respect whether the conclusion can be generalised. That in mind, Bush et al. 2018 contains two different datasets sampling birth records from the English and Welsh population, of which the present work uses only one of them (from Table S15; the ONS dataset, which is much greater in depth but narrower in temporal scope). The second dataset – which has the same popularity and diversity indicators – is available in Table S6, and may be viewed as a representative population sample from 1838-2014, albeit of varying coverage depths per year. Nevertheless, a cursory examination of this data suggests that, as with the ONS data, there is a strongly negative correlation of popularity and diversity for the years 1996-2010 (this time period chosen because within it there are > 10,000 birth records per year). If we accept this as independent support of the conclusion drawn using the ONS data (and thereby the utility of this dataset), then it may be worth looking at how popularity and diversity correlate throughout history, too. This is of particular relevance as the conclusion drawn in the abstract (“we can predict one indicator from the other indicator”) implies a general relationship between the concepts that may not be universally true and that instead they may only “consistently measure the same concept” for a limited period of time.***

***I have expanded the Table S6 dataset substantially since the original publication; see Bush 2024 (https://doi.org/10.5195/names.2024.2543) and its raw data, specifically https://github.com/sjbush/uk_bmd/blob/main/dataset_B/summary_of_records_per_year.txt, which is essentially equivalent to Table S15 and contains the columns “most popular forename (% of records)” and “forename diversity (i.e., ratio of the no. of different forenames to the total no. of records per year)”. It should be clear from this data that before (approx.) the second half of the 20th century, and compared to contemporary records, forename diversity was not only persistently lower but that popular names were given to a higher proportion of people and remained popular for longer, e.g. John and Mary were consistently the most popular male and female names, respectively, for a century (see https://demos.flourish.studio/namehistory/?names=John,Mary, which visualises data from Bush et al. 2018). So, although the present work (using late-20th century data) finds that one indicator predicts the other, it also appears the case that (earlier in the 20th century) popularity could vary without discernible change to diversity. Consequently, the two variables do not necessarily change simultaneously. This may be the subject of a more in-depth analysis but could be usefully commented upon here.***

 Thank you for your valuable comment. Regarding the review paper, the two points (“names have become less popular over time” and that “raw data on names is restricted from being openly shared, making it common for only the ranking of popular names”) are supported not only by the review paper but also by other references I include (Bao et al., 2021; Bush, 2020; Bush et al., 2018; Gerhards & Hackenbroch, 2000; Kuipers & Askuri, 2017; Mignot, 2022; Ogihara, 2021, 2022; Ogihara & Ito, 2022; Ogihara et al., 2015; Twenge et al., 2010, 2016). Moreover, whether the review paper is made openly available would not directly affect the decision on this paper.

 I also appreciate your second point. As you already acknowledged, it is unclear how the second dataset is representative (“The second dataset – which has the same popularity and diversity indicators – is available in Table S6, and may be viewed as a representative population sample from 1838-2014, albeit of varying coverage depths per year.”). In the original paper (Bush et al., 2018), the sample size for each year in the dataset is not sufficiently large and its representativeness for each year is not explained (“This approximates 130,000 to 230,000 records per year from 1838–1950, 25,000 to 100,000 records per year from 1951–2000, and 5000 to 15,000 records per year from 2001 to 2014”; p. 3). In contrast, the sample sizes are sufficiently large and representativeness of the newer dataset that I analyzed is apparently high. Thus, I analyzed the newer dataset and did not analyze the older dataset.

***Finally, could some comment also be made on the gender of names, and the effect this may have on the conclusions? In contemporary UK name data, there is typically a larger pool of female than male names (discussed in Bush 2020, https://doi.org/10.1080/00277738.2020.1775471): that is, one would expect female names to have a higher “diversity indicator” and lower “popularity indicator” than male names. Accordingly, a direct correlation of popularity with diversity may not quite be comparing like with like: diversity being affected more strongly by female names, but popularity by male (such that in Table S15, the top 1 names, across the whole population, are all male).***

 I appreciate your comment. The original study (Bush et al., 2018) did not analyze name trends by gender, so this study did not either. However, I agree with your point. Thus, I have added this as a limitation, as follows.

 “This study analyzed the dataset yielded by the past study (Bush et al., 2018), which did not distinguish between boys’ and girls’ names. Although a different pattern is not predicted based on gender, it is desirable to investigate the relationship between the diversity and popularity of names for boys and girls separately in the future.” (Limitation and future direction section)

 Thank you for your further consideration of this manuscript.

 I look forward to hearing from you at your earliest convenience.

 Sincerely,

 Yuji Ogihara, Ph.D.

 Aoyama Gakuin University

 Department of Psychology, College of Education, Psychology and Human Studies

 Address: 4-4-25 Shibuya, Shibuya-ku, Tokyo, 150-8366, Japan

 E-mail: yogihara@ephs.aoyama.ac.jp

 Web: https://sites.google.com/site/yujiogiharaweb/english

# Reviewer report: Reviewer response for version 1

**DOI:** 10.5256/f1000research.178684.r378190

**Published:** 2025-05-06

**Authors:** Han-Wu-Shuang Bao

**Affiliations:** 1 School of Psychology and Cognitive Science, East China Normal University, Shanghai, Shanghai, China

In this Brief Report, the author performed a simple correlation analysis with Bush et al.’s (2018) published dataset on UK first names, showing that when the most popular names were more often used, there were also more diverse types of unique names. I think the author has addressed one of the important issues in name research and cultural change research, which lacked an empirical test before. While I believe the main argument that name popularity and name diversity can predict each other is worth publishing, I have several suggestions that may improve the strength of this argument.

 First, the analysis presented in this report was simple correlation over time, which might be spurious due to covariance among year, popularity, and diversity. To more rigorously test the key relationship, I suggest either controlling for year or conducting a cross-correlation analysis and a Granger causality test. The latter two methods are usually preferred for more rigorous time series analysis in cultural change research. In doing so, the author could examine whether popularity precedes diversity, diversity precedes popularity, or they shift just concurrently. It becomes more important here because such high correlations found in this report might simply due to the fact that any linear trends over time would be highly correlated (positively or negatively) with each other.

 Second, to partial out the confound by a common linear trend, I also suggest performing a simulation analysis. Specifically, you can generate or resample (e.g., bootstrap) large random samples of “names”, with each sample consisting of a sufficient number of “names” simulating a sample of names in a “year”. Then, for each sample, you can calculate the indices of popularity and diversity. Finally, across these simulated samples, you can test the correlation between popularity and diversity. In this way, you can test this relationship without the time confound. This may also answer if there is a purely statistical relationship between popularity and diversity, providing a better understanding of the main research question here.

 Besides these suggestions, I have one minor comment on including more past evidence for name diversity. Bao et al. (2021) indeed tested changes in six name indices in China, where “standard deviation of name length” and “proportion of three-character given names” can both be regarded as indices of name diversity in Chinese naming practices. So I suggest adding them as another piece of evidence into the section “Diversity of names has increased”.

Is the work clearly and accurately presented and does it cite the current literature?

Yes

If applicable, is the statistical analysis and its interpretation appropriate?

No

Are all the source data underlying the results available to ensure full reproducibility?

Yes

Is the study design appropriate and is the work technically sound?

No

Are the conclusions drawn adequately supported by the results?

Partly

Are sufficient details of methods and analysis provided to allow replication by others?

Yes

Reviewer Expertise:

cultural change, names, naming practices, name uniqueness

I confirm that I have read this submission and believe that I have an appropriate level of expertise to confirm that it is of an acceptable scientific standard, however I have significant reservations, as outlined above.

# Response

**Published:** 2025-07-06

**Authors:** Yuji Ogihara

**Affiliations:** Psychology, Aoyama Gakuin Daigaku, Shibuya, Tokyo, Japan

July 6th, 2025

 Dear Dr. Han-Wu-Shuang Bao,

 Thank you very much for reviewing my manuscript and providing valuable comments.

 I have modified the manuscript extensively according to the reviewers’ comments.

 I offer my responses to each comment below.

 I have copied and pasted all of your comments without making changes.

***In this Brief Report, the author performed a simple correlation analysis with Bush et al.’s (2018) published dataset on UK first names, showing that when the most popular names were more often used, there were also more diverse types of unique names. I think the author has addressed one of the important issues in name research and cultural change research, which lacked an empirical test before. While I believe the main argument that name popularity and name diversity can predict each other is worth publishing, I have several suggestions that may improve the strength of this argument.***

 Thank you for your words of praise. Following your comments below, I have modified the manuscript.

***First, the analysis presented in this report was simple correlation over time, which might be spurious due to covariance among year, popularity, and diversity. To more rigorously test the key relationship, I suggest either controlling for year or conducting a cross-correlation analysis and a Granger causality test. The latter two methods are usually preferred for more rigorous time series analysis in cultural change research. In doing so, the author could examine whether popularity precedes diversity, diversity precedes popularity, or they shift just concurrently. It becomes more important here because such high correlations found in this report might simply due to the fact that any linear trends over time would be highly correlated (positively or negatively) with each other.***

 Thank you for your valuable comment. I agree with you that it is important to consider this point carefully.

 I do not claim that there is a causality between the two indicators. As I write in the article, it is important to predict one indicator from the other indicator in name research. It is not necessary to determine whether popularity precedes diversity, diversity precedes popularity, or they shift just concurrently. Thus, I think simple correlations are sufficient, at least in this context. Nevertheless, I also think that the analyses you suggested work well in cultural change research.

 However, if you do not find my response to be sufficient, I will reconsider this point. I would appreciate it very much.

***Second, to partial out the confound by a common linear trend, I also suggest performing a simulation analysis. Specifically, you can generate or resample (e.g., bootstrap) large random samples of “names”, with each sample consisting of a sufficient number of “names” simulating a sample of names in a “year”. Then, for each sample, you can calculate the indices of popularity and diversity. Finally, across these simulated samples, you can test the correlation between popularity and diversity. In this way, you can test this relationship without the time confound. This may also answer if there is a purely statistical relationship between popularity and diversity, providing a better understanding of the main research question here.***

 I appreciate your constructive comment. That is a fascinating idea. I agree that such a simulation analysis can contribute to a better understanding of names and naming practices.

 In contrast, I think that the approach is beyond the scope of this article which analyzes the actual data from the United Kingdom.

 Moreover, this simulation could tend to lack ecological validity. The results should strongly depend on parameters of a hypothetical and imaginary dataset.

 Furthermore, this simulation lacks a time perspective. Our focus is on how names (and name indicators) change over time.

 Therefore, I would like to consider this new idea in a different study. Anyway, thank you very much for your important input.

***Besides these suggestions, I have one minor comment on including more past evidence for name diversity. Bao et al. (2021) indeed tested changes in six name indices in China, where “standard deviation of name length” and “proportion of three-character given names” can both be regarded as indices of name diversity in Chinese naming practices. So I suggest adding them as another piece of evidence into the section “Diversity of names has increased”.***

 Thank you for your comment. I agree that the indicators you mention are related to name diversity. Yet, these indicators do not directly show name diversity because they focus on “name length.” Even when the standard deviation of name length increases, name diversity can decrease. For example, even when names consisting of one or three characters increase, if the same names are given, diversity can decrease. Therefore, I maintain this part.

 Thank you for your further consideration of this manuscript.

 I look forward to hearing from you at your earliest convenience.

 Sincerely,

 Yuji Ogihara, Ph.D.

 Aoyama Gakuin University
